# A new family of phosphoinositide phosphatases in microorganisms: identification and biochemical analysis

**DOI:** 10.1186/1471-2164-11-457

**Published:** 2010-08-02

**Authors:** Nicola J Beresford, Charis Saville, Hayley J Bennett, Ian S Roberts, Lydia Tabernero

**Affiliations:** 1 Faculty of Life Sciences, Michael Smith Building, University of Manchester, Manchester, M13 9PT, UK; 2 Current Address: Mycobacterial Research, National Institute for Medical Research, The Ridgeway, Mill Hill, London, NW7 1AA

## Abstract

**Background:**

Phosphoinositide metabolism is essential to membrane dynamics and impinges on many cellular processes, including phagocytosis. Modulation of phosphoinositide metabolism is important for pathogenicity and virulence of many human pathogens, allowing them to survive and replicate in the host cells. Phosphoinositide phosphatases from bacterial pathogens are therefore key players in this modulation and constitute attractive targets for chemotherapy. MptpB, a virulence factor from *Mycobacterium tuberculosis*, has phosphoinositide phosphatase activity and a distinct active site P-loop signature HCXXGKDR that shares characteristics with eukaryotic lipid phosphatases and protein tyrosine phosphatases. We used this P-loop signature as a "diagnostic motif" to identify related putative phosphatases with phosphoinositide activity in other organisms.

**Results:**

We found more than 200 uncharacterised putative phosphatase sequences with the conserved signature in bacteria, with some related examples in fungi and protozoa. Many of the sequences identified belong to recognised human pathogens. Interestingly, no homologues were found in any other organisms including Archaea, plants, or animals. Phylogenetic analysis revealed that these proteins are unrelated to classic eukaryotic lipid phosphatases. However, biochemical characterisation of those from *Listeria monocytogenes *and *Leishmania major*, demonstrated that, like MptpB, they have phosphatase activity towards phosphoinositides. Mutagenesis studies established that the conserved Asp and Lys in the P-loop signature (HCXXG**KD**R) are important in catalysis and substrate binding respectively. Furthermore, we provide experimental evidence that the number of basic residues in the P-loop is critical in determining activity towards poly-phosphoinositides.

**Conclusion:**

This new family of enzymes in microorganisms shows distinct sequence and biochemical characteristics to classic eukaryotic lipid phosphatases and they have no homologues in humans. This study provides a foundation for examining the biological role of this new family of phosphatases and their potential as pharmaceutical targets against infectious diseases.

## Background

Phosphatidylinositols are important metabolites present in animal cell membranes. They can be phosphorylated at three main positions of the inositol head (D-3, 4, and 5) to generate seven different phosphoinositides (PIs). Their metabolism is tightly controlled by phospholipases that in some instances generate second messengers such as inositol 1,4,5-trisphosphate (Ins(1,4,5)P3) and diacylglycerol (DAG). In addition, kinases and phosphatases control the levels of the mono, di- or tri-phosphorylated forms [1-5]. PI phosphatases belong to different families of enzymes; PTEN and myotubularin inositol 3-phosphatases (3-ptases) belong to the protein tyrosine phosphatase (PTP) superfamily and have both protein and lipid phosphatase activities [6,7]. Inositol 4-phosphatases (4-ptases), share with PTPs the conserved active site signature CX_5_R (P-loop motif) where the Cys is the nucleophile in catalysis and Arg binds the phosphate group in the substrate. The inositol 5-phosphatases (5-ptases) are Mg^2+^-dependent enzymes related to endonucleases (reviewed in [6,7]).

PIs are implicated in the regulation of a wide variety of cellular functions, including signal transduction, membrane dynamics, cytoskeleton arrangements, endocytosis, endosome trafficking, permeability and transport across membranes [1,3]. PIs are also important in facilitating phagocytosis of pathogenic bacteria and the subsequent phagosomal maturation leading to bacteria decay. This process is mediated by PI(3)P and PI(3,5)P2 [4]. Pathogenic bacteria have evolved sophisticated strategies to escape the innate immune response, using PI metabolising proteins such as phosphatases and kinases to block phagosome maturation. Some of these bacterial phosphatases like SigD/SopB, IpgD and SapM have phosphoinositide activity resulting in the alteration of PI levels in the host membranes [8-10].

*Mycobacterium tuberculosis *(*Mtb*) is an exceptionally successful pathogen that is able to survive in the host for a lifetime. *Mtb *enters macrophages in a phosphoinositide-3-kinase (PI3K) dependent manner and uses lipid analogues (LAM) [11] and PI metabolising enzymes (SapM [12]) to keep low levels of PI(3)P that prevent recruitment of Rab7 and phagosomal maturation. MptpB is a secreted phosphatase [13] critical for *Mtb *survival in host cells [14]. We have recently shown that it has protein and PI phosphatase activity [15] and could act together with SapM to control PI(3)P levels in the host.

MptpB has a unique active site P-loop motif (160-167): HCFAG**KD**R, which is strictly conserved in other mycobacterial species, where the Asp165 is an essential catalytic residue and Lys164 is important for PI binding [15]. This signature shares similarities with that of eukaryotic 3-ptases myotubularin (MTM), HCSDGW**D**R, and PTEN, HC**K**AG**K**GR, despite a low overall sequence homology (10.6% for MTMR2 and 9.8% for PTEN). The MTM/PTEN family of 3-ptases is conserved in mammals, *Drosophila melanogaster, Caenorhabditis elegans, Arabidopsis thaliana*, yeast, kinetoplastids and several protozoa [16-20]. However, homologous 3-ptases have not been identified in bacteria. Instead, some pathogenic bacteria have multifunctional 5-ptases such as SopB from *Salmonella *and *IpgD *from *Shigella *[21]. The P-loop features and catalytic profile of MptpB, together with the lack of MTM and PTEN orthologues in bacteria suggested that MptpB like phosphatases may constitute an alternative type of PI phosphatase in prokaryotes. This hypothesis prompted us to further investigate the existence of MptpB related protein sequences in other organisms.

In this study, bioinformatics explorations revealed a large family of MptpB related sequences present mostly in bacteria, with some in fungi and protozoa. Several of the sequences identified are found in recognised intracellular pathogens. No MptpB homologues were found in Archaea, plants or animals. We characterised biochemically, selected proteins from different organisms (two bacterial and one from a protozoa) and confirmed that they have phosphatase activity towards both phosphorylated peptides and PIs. Mutagenesis of conserved P-loop residues confirmed a Cys based mechanism of catalysis, where the conserved Asp is an essential catalytic residue. Furthermore, we provide evidence for the importance of key residues in the P-loop as determinants in PI substrate specificity. Substitution of P-loop residues to mimic that of eukaryotic 3-ptases, resulted in mutant enzymes with enhanced phosphatase activity and new poly-phosphate PI specificity.

Phylogenetic analysis shows that this new family of PI phosphatases is not related to the classic eukaryotic lipid phosphatases like MTMs/PTEN. Moreover, these phosphatases exhibit distinct and unusual features in their catalytic profile, sequence and domain organisation to other known PI and inositol phosphatases, for which we designated them as atypical lipid phosphatases (ALPs).

## Results

### Bioinformatics analysis reveals a large family of MptpB related sequences

Initial sequence homology searches were done using the full-length MptpB sequence. These searches found 81 protein sequences in bacteria and one single sequence from fungi (*Coprinopsis cinerea okayama*) (Table 1). No sequences were found in Archaea, plants or animals. Some bacterial species contained more than one gene, like *Listeria monocytogenes, Lactobacillus brevis *and *Rhodopseudomonas palustris*. These 81 proteins are still uncharacterised functionally and one third of them are annotated as hypothetical proteins. The remaining are annotated as dual-specificity phosphatases (DSPs) (41%) or PTPs (21%) based on the presence of the CX_5_R motif, although there is not experimental evidence in the literature to support this assignment.

**Table 1:** Classification of sequences identified through Blast analysis and ScanProsite

*Organism*	*Blast search*	*ScanProsite search*
**Bacteria**	**80**	**163**
Firmicutes	15	56
Proteobacteria	34	60
Actinobacteria	26	40
Bacteroidetes	4	2
Chloroflexi	1	2
Cyanobacteria (*Nostoc commune*)		1
Deinoccoccus-Thermus		**1**
Verrucomicrobia (*Opitutaceae*)		**1**
**Eukaryota**	**1**	**46**
Fungi	1	40
Parabasalidea (*Trichomonas vaginalis*)		1
Trypanosomatide (*Leishmania*)		4
Metazoa (*C.elegans*)		1

Sequences were identified in both Gram positive (48%) and Gram negative (51%) bacteria. The MptpB P-loop signature is conserved in all sequences, except 7, of which the Cys residue is absent in 3 sequences (2 from *Burkholderia phytofirmans *and 1 from *Mycobacterium paratuberculosis*) and the Asp residue is absent in 4 sequences (*Bacteroides ovatus, Bacteroides uniformis, Bacteroides fragilis, Bacteroides thetaiotaomicron*). As both residues are important for catalysis [15] these 7 sequences are probably inactive phosphatases and were excluded in subsequent analyses.

**Figure 1:**
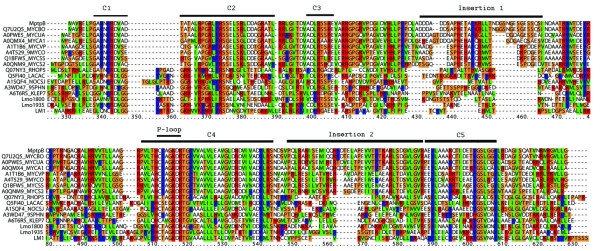
**Alignment of MptpB related sequences**. Alignment of MptpB related sequences identified from a Blast analysis with one representative sequence from each group in the phylogenetic tree and the sequences of the proteins characterised biochemically in this study, Lm800, Lm1935 and LM1 (the complete alignment is in the Additional file 1). The alignment highlights that the sequences are highly divergent except from the phosphate-binding loop (P-loop) in the active site region with an extended signature (C4). There are additional regions of high conservation (labelled C1-C5). Insertions 1 and 2 are unique to the mycobacteria sequences. Alignment prepared using ClustalX [35].

A multiple sequence alignment of the bacterial ALPs, highlights five main regions of conservation in the family (C1-C5 in Figure 1 and additional file 1). The active site region shows the highest conservation, with the P-loop: HCXXGKDR[TA]G, and a contiguous extended signature, reminiscent of that found in DSPs [22] (C4 in Figure 1). The P-loop motif is the landmark feature of this family of sequences and retains the unusual composition found in MptpB with the strictly conserved Lys and Asp, suggesting likely similarities in catalytic activity. The four additional regions, C1-C3 and C5, show good conservation (30-55%) with some residues showing greater than 50% conservation, in agreement with those regions forming part of the main structural core of the phosphatase domain. Although the P-loop shares the CX_5_R signature with PTPs, other key functional motifs in PTPs are not present in the ALPs sequences. These include the WPD motif, which contains the catalytic Asp in PTPs, the KNRY motif (pTyr binding motif) and the Q-loop, characteristic of tyrosine specific phosphatases [23]. The absence of the WPD loop suggests that an alternative Asp residue in ALPs may function as the cognate general acid in the dephosphorylation reaction. The most likely candidate is the conserved Asp in the P-loop, which is a catalytic residue in MptpB [15]

**Figure 2:**
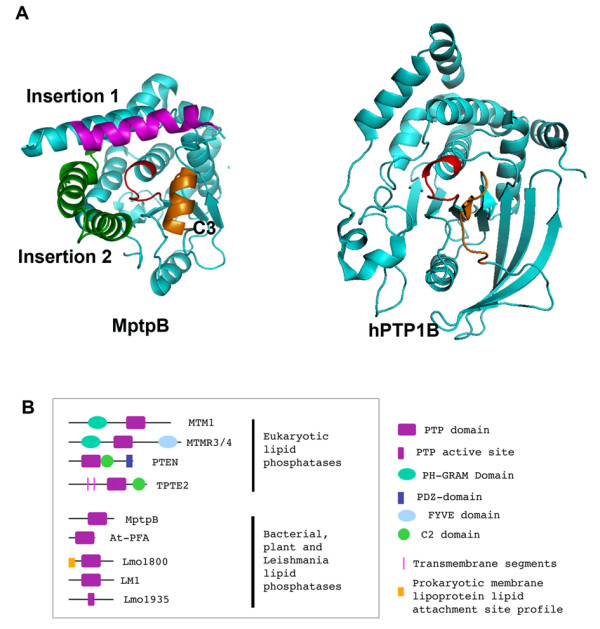
**A. Unique structural features in mycobacterial ALPS**. The structure of MptpB, left (PDB ID 2OZ5) is shown as a ribbon diagram. The mycobacterial insertion 1 (magenta) and insertion 2 (green) are shown together with the alpha helix (orange) conserved in ALPs (C3). The structure of human PTP1B (PDBID 1AAX) is shown on the right. In orange is the analogous region to C3, which adopts a non-helical conformation in PTPs. The active site P-loop is shown in red. Ribbon diagrams generated using PyMol (DeLano Scientific LLC). **B**. **Domain organisation in lipid phosphatases**. PTEN and MTM type lipid phosphatases have a phosphatase PTP domain and additional lipid binding domains like PH-GRAM, C2, FYVE that exert allosteric control and anchor the PTP domain to different membrane locations. ALP and PFA lipid phosphatases lack the classic lipid binding domains, only Lmo1800 contains a predicted lipid attachment sequence.

The alignment of ALPs highlighted that mycobacterial sequences contain two unique insertions flanking the active site motif: insertion 1 (residues 85-117 in MptpB) and insertion 2 (197-235 in MptpB) (Figure 1 and Figure 2A). In *M. tuberculosis *and *M. bovis *there is a single residue alteration in insertion 1, while the corresponding region in other mycobacterial species is also unique but unrelated. Insertion 2 also has deviations between *M. tuberculosis *and *M. bovis *sequences and the remaining mycobacterial sequences. These insertions appear to be important for functional regulation of MptpB as suggested from the crystallographic structures. Insertion 2 forms a lid that covers the active site and could control substrate access (Figure 2A) [24], and insertion 1 changes conformation upon ligand binding [25]. Another distinct structural feature of MptpB is an alpha helix in the conserved motif C3 (Figure 2A), which is not present in the structures of PTPs (including human PTP1B) or DSPs. This helix provides a secondary binding pocket for inhibitory compounds [25,26] and may be important in conferring selectivity. The C3 motif is relatively conserved in the ALP family with a high proportion of basic and acidic residues throughout, suggesting a good potential for helical content in this region.

The domain organisation of this new family shows a main difference with other eukaryotic lipid phosphatases, which generally contain a number of characteristic modules for membrane targeting and lipid binding (PH, C2, GRAM, FYVE). In contrast, ALPs contain the phosphatase domain only and lack any classic lipid binding modules (Figure 2B), suggesting that they use alternative mechanisms for lipid binding and membrane anchoring, maybe mediated by effector proteins.

### Phylogenetic analysis of MptpB related sequences

The occurrence of one single eukaryotic sequence in the blast search was intriguing, but the high conservation of the P-loop motif, suggested that perhaps other eukaryotic sequences may still retain this signature albeit with a low overall homology for the rest of the sequence. To test this, we carried out a ScanProsite search using the active site motif "HCXXGKDR". The search returned a total of 209 sequences, of which 163 were bacterial and 46 were from lower eukaryotes (Table 1). The majority of eukaryotic sequences identified were fungi (40), with 4 *Leishmania *sequences (2 *L. infantum *and 2 *L. major*), 1 sequence from *Caenorhabditis elegans *and 1 sequence from *Trichomonas vaginalis*. A total of 31% of the sequences identified in ScanProsite were also present in the Blast hit list, indicating that in the remaining sequences the overall sequence identity was less than 30% to MptpB. Interestingly, more than 30 sequences of the total list belong to pathogens.

Other known bacterial lipid and inositol phosphatase sequences were missing from our hit list. This is the case of SopB/SigD and IpgD, with a slight different motif: **N**CKSGKDR, and low sequence identity to MptpB (~11%). Also absent are the mammalian 4-ptases (with the motif XCKS**A**KDR) and 5-ptases that lack the CX_5_R consensus motif.

**Figure 3:**
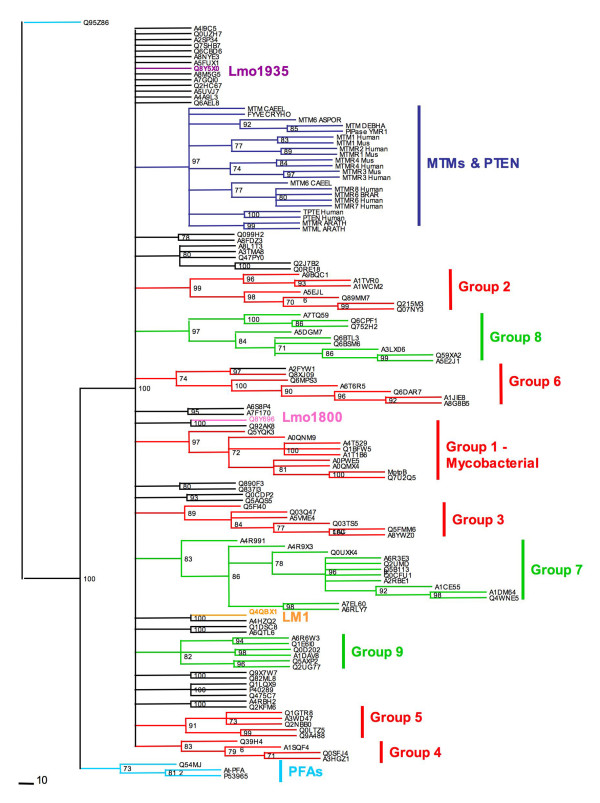
**Phylogenetic tree of MptpB related phosphatases with eukaryotic lipid phosphatases**. An unrooted phylogenetic tree was constructed using the results from the BLAST analysis, the eukaryotic sequences identified in ScanProsite and reference sequences including eukaryotic lipids phosphatases (myotubularins, PTEN and PFAs). The bacterial MptpB related sequences (red) cluster independently of eukaryotic lipid phosphatases (labelled MTMs and PTEN in dark blue) and atypical lipid phosphatases PFAs (cyan). Sequences in red show the six bacterial groups formed by sequences identified in Blast, while sequences in green show the three groups of eukaryotes with MptpB-like motif. Generated using RAxML and Treeview [37,38] with bootstrap values indicated.

A phylogenetic tree was compiled using the bacterial ALP sequences from the Blast search together with eukaryotic sequences from the ScanProsite motif search, the phosphatase domain of classical eukaryotic lipid phosphatases (MTM, PTEN) and the recently reported PIP3 phosphatases from plants and kinetoplastids PFAs [27]. The analysis identified eleven clusters (Figure 3 and Additional file 2), with MTMs, PTEN sequences (dark blue in Figure 3) and PFAs, (cyan in Figure 3) clustering independently from the ALP sequences. The bacterial and eukaryotic ALPs form separate clusters with bacterial sequences in groups 1-6 (red in Figure 3) and eukaryotic sequences in groups 7-9 (green in Figure 3). All mycobacterial sequences cluster together in group 1 reflecting a highest homology within this subgroup. Gram positive (groups 1 and 3) and Gram negative (groups 2, 5, 6) bacteria group separately with the exception of group 4, which contains a mix of Gram positive and negative bacteria. Group 8 is formed by yeast species, while the other groups contained a mix of species. Only *Aspergillus nidulans *has sequences in different groups (group 7 and group 9). A number of sequences fail to cluster into any group (black in Figure 3), but remain independent of classic eukaryotic lipid phosphatases and PFAs. Overall the phylogenetic tree emphasises the diversity of the APL sequences with clear separation between the bacterial and fungal/protozoan clusters and confirms that none of them are closely related to classical eukaryotic 3-ptases typified by MTMs and PTEN.

### MptpB related sequences exhibit both protein phosphatase and lipid phosphatase activity

We previously reported that MptpB had both protein phosphatase and lipid phosphatase activity. We also established that Cys160, Lys164, Asp165 and Arg166 in the P-loop, are important residues involved in catalysis or substrate binding [15]. The conservation of these residues in the ALP sequences, suggested they could have a similar enzymatic profile, which could be relevant to PI metabolism. To test this hypothesis, two prokaryotic sequences and one eukaryotic sequence from our hit list were selected for enzymatic analysis, all of them belonging to intracellular pathogens; Lmo1800 and Lmo1935 from *L*. *monocytogenes *and LM1 from *L. major*. These sequences occupy different locations within the phylogenetic tree (Figure 3) and had sequence identities of only 22-24% to MptpB. Despite their relatively poor sequence homology the P-loop motif is almost identical to MptpB with only one substitution of F for T in position two.

**Figure 4:**
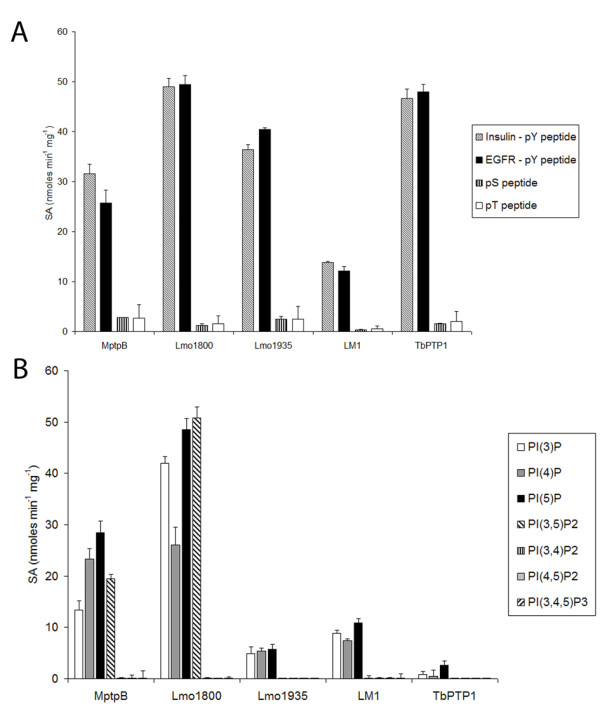
**MptpB and related phosphatases have both protein phosphatase and lipid phosphatase activity**. (**A**) Specific activity (SA) for His-MptpB WT, Lmo1800 WT, Lmo1935 WT, His-LM1 and His-TbPTP1 [15] towards phosphorylated peptide substrates tested. (**B**) Specific activity of the same proteins towards PI substrates is shown. None of the phosphatases had activity on PI(3,4)P2, PI(4,5)P2 or PI(3,4,5)P3. Error bars indicate SEM.

Biochemical analyses confirmed that all three proteins had phosphatase activity, with the ability to dephosphorylate the generic phosphatase substrate pNPP. Lmo1800 shows the highest specific activity (SA) of 63.4 nmoles min^-1 ^mg^-1^, followed by Lmo1935 of 57.5 nmoles min^-1 ^mg^-1 ^and LM1 of 45.5 nmoles min^-1 ^mg^-1^, comparable to MptpB of 46.9 nmoles min^-1 ^mg^-1 ^and the control protein phosphatase, *Tb*PTP1, 53.8 nmoles min^-1 ^mg^-1^. In addition, they all dephosphorylate p-Tyr peptides, with Lmo1800 showing the greatest SA of 49.4 nmoles min^-1 ^mg^-1^, whilst LM1 showed considerably lower activity, 12.1 nmoles min^-1 ^mg^-1 ^(Figure 4A). Dephosphorylation of p-Ser and p-Thr peptides was significantly lower for all phosphatases, with MptpB having the highest SA of 2.82 nmoles min^-1 ^mg^-1^, and LM1 the lowest at 0.58 nmoles min^-1 ^mg^-1 ^(Figure 4A). The dephosphorylation ratio of pY: pS/T peptides for MptpB and Lmo1935 is 11 : 1 and 16 : 1, respectively, comparable to the DSPs such as VHR (2.9 : 1), in contrast to Tyr specific PTPs like hPTP1B that show a ratio greater than 1.48 × 10^5 ^: 1 [28]. Based on the results it appears that Lmo1800 and LM1 have a stronger preference for p-Tyr peptides while Lmo1935 exhibits a similar activity profile for the phospho-peptides to MptpB.

We further tested if these proteins had phosphatase activity towards PIs, as the P-loop signature seem to predict. We tested all seven PI forms and we found that the control protein tyrosine phosphatase, *Tb*PTP1, had little activity of towards any of the PIs (< 2.6 nmoles min^-1 ^mg^-1^). Lmo1800 exhibited high activity towards PI(3,5)P2 (50.8 nmoles min^-1 ^mg^-1^) and mono-phosphate PIs PI(3)P (41.9 nmoles min^-1 ^mg^-1^), PI(4)P (26.1 nmoles min^-1 ^mg^-1^) and PI(5)P (48.6 nmoles min^-1 ^mg^-1^), with no detectable activity towards the remaining PIs (Figure 4B). Both Lmo1935 and LM1 show also activity towards mono-phosphorylated PIs (ranging from 4.9-5.8 nmoles min^-1 ^mg^-1 ^and 8.2-10.9 nmoles min^-1 ^mg^-1^, respectively) but not for PI(3,5)P2 (Figure 4B). Although the activity of LM1 towards PIs is lower than MptpB, it was of a similar magnitude to p-Tyr peptides indicating no preferred substrate. This is in contrast to Lmo1935, which has a stronger preference for peptide substrates (7-fold increase). None of the phosphatases were able to dephosphorylate contiguous di-phosphate PIs: PI(3,4)P2, PI(4,5)P2 and the tri-phosphate PI(3,4,5)P3, showing a similar catalytic profile to MptpB [15]. Other phosphoester substrates were tested and confirmed the preference of MptpB and Lmo1800 for the p-Tyr amino acid over p-Ser and p-Thr and showed modest activity for 5'AMP and 5'IMP (Table 2).

**Table 2:** Activity of ALPs on phosphosubstrates.

	**Specific Activity (nmoles PO**_**4**_**/min/mg)**			
**Substrate**	**MptpB**	**LM1**	**Lmo1935**	**Lmo 1800**

pNPP	46.94 ± 0.31	45.47 ± 1.52	57.47 ± 0.96	63.38 ± 0.71
5' AMP	2.89 ± 0.55	0.34 ± 0.26	0.48 ± 0.02	6.71 ± 0.43
5' IMP	3.35 ± 0.16	N.D	0.35 ± 0.03	3.90 ± 0.66
P-Choline	0.63 ± 0.09	N.D	0.26 ± 0.05	0.58 ± 0.16
P-Ethanolamine	0.51 ± 0.23	N.D	0.2 ± 0.02	0.37 ± 0.14
Glycerol 2-phosphate	0.95 ± 0.26	N.D	0.12 ± 0.03	1.07 ± 0.57
P-Ser	1.0 ± 0.12	3.17 ± 1.36	0.57 ± 0.21	N.D
P-Thr	0.69 ± 0.05	N.D	0.42 ± 0.04	0.78 ± 0.34
P-Tyr	6.48 ± 0.40	N.D	0.54 ± .04	9.68 ± 3.29
NaP	1.24 ± 0.07	N.D	0.45 ± 0.1	0.15 ± 0.05

Dephosphorylation of pNPP is shown as a reference.

N.D, no activity detected.

### P-loop composition in lipid phosphatases: identification of determinants for substrate specificity

**Figure 5:**
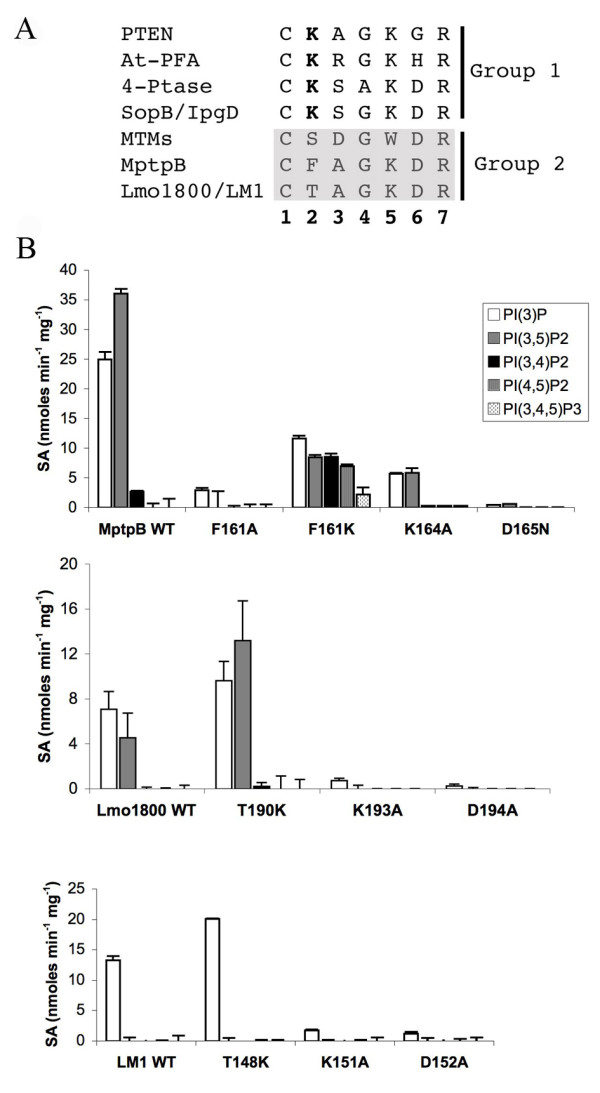
**A. P-loop composition of different lipid phosphatase activity**. Two groups of lipid phosphatases can be assigned based on the different amino acid composition of the active site P-loop. Group 1 contains 3 or more basic residues and a conserved K in position two, while Group 2 have 1 or 2 basic residues and none in position 2**. B. Effect of mutagenesis of P-loop residues on the catalytic profile**. Specific activity (SA) for WT and mutants of MptpB, Lm1800 and LM1 enzyme using different phosphoinositides as substrates. Addition of a second basic residue in the P-loop increases activity towards di-phosphorylated PIs and results in new activity for MptpB towards PI(3,4,5)P3. Error bars indicate SEM.

Comparison of the P-loop composition between different PI phosphatases reveals two distinct groups. Group 1, with PTEN, *Arabidopsis thaliana *PFA (At-PFA), 4-ptases and SopB/IgpD, which have three or more basic residues, and group 2, with MptpB, MTMs, Lmo1800 and LM1, that have only one or two basic residues and the conserved catalytic Asp in position six (Figure 5A). All of them, except MTMs, have a conserved Lys residue in position 5, important in substrate binding [15,29]. Group 1 enzymes have an additional Lys in position 2, which appears to correlate with the ability to dephosphorylate PI(3,4,5)P3 and poly-phosphoinositols. Group 2 enzymes lack this extra Lys and are unable to dephosphorylate PI(3,4,5)P3 and most di-phosphorylated PIs, favouring instead mono-phosphorylated PI substrates [15,30]. The absence of this extra basic residue, presumably important to compensate for additional negative charge in poly-phosphate substrates, may account for the lack of activity towards those PIs in group 2. To test this hypothesis and to corroborate the functional role of the conserved Lys and Asp at positions 5 and 6 we produced a number of P-loop single mutants. We previously established the function of Asp165 in MptpB as an essential residue in catalysis [15]. Mutagenesis of the homologue Asp residue (Asp194 and Asp152) in Lmo1800 and LM1 to Ala resulted in a significant loss in activity, with < 10% of WT activity (Figure 5B).

Another residue in the P-loop, Lys164 in MptpB, is involved in substrate binding and particularly important for dephosphorylation of PI(3,5)P2 [15]. Mutation of the cognates Lmo1800-Lys193 and LM1-Lys151 to Ala resulted in a significant loss in activity towards PI(3,5)P2 but retained 10% of the WT activity for PI(3)P (Figure 5B).

Having established the importance of residues in position 5 and 6, we then wanted to explore the importance of Lys in position 2 in substrate specificity. We mutated position 2 residues in MptpB and related enzymes to Lys to see if we could convert a group 2 lipid phosphatase into a group 1 phosphatase with similar substrate specificity. We found that in MptpB, the residue in position 2, Phe161, when mutated to Lys, results in an mutant enzyme with new activity for di-phosphorylated substrates PI(3,4)P2 (8.5 nmoles min^-1 ^mg^-1^) and PI(4,5)P2 (7.0 nmoles min^-1 ^mg^-1^) and even shows activity towards the tri-phosphorylated PI(3,4,5)P3 (2.2 nmoles min^-1 ^mg^-1^) (Figure 5B). Note that the WT enzyme does not dephosphorylate any of these substrates.

Mutation of the equivalent position in Lmo1800 (Thr190) to Lys resulted in increased dephosphorylation of PI(3)P (1.4-fold), a 3-fold increase of activity towards and PI(3,5)P2 with low activity for PI(3,4)P2 (0.2 nmoles min^-1 ^mg^-1^). For LM1, mutation of Thr148 to Lys, also shows an increase (1.5-fold) in activity for PI(3)P but not towards di- or tri-phosphorylated PIs, suggesting that other factors and possibly conformational arrangements in the active site of Lmo1800 and LM1 determine substrate preference.

## Discussion

In this study, we have used the P-loop signature of the bacterial lipid phosphatase MptpB as a "diagnostic motif" to identify related putative lipid phosphatases. We found a whole new family of predicted phosphatases in bacteria, with some instances in fungi and protozoa. Interestingly, no homologues were found in other organisms such as Archaea, plants or animals. These proteins have not previously been characterised functionally and at least one third of them are not annotated. The existence of such a large family of putative lipid phosphatases in bacteria is unexpected. Very few bacteria, with the exception of actinobacteria, produce inositols or phosphoinositides, therefore they generally lack the enzymes involved in inositol metabolism [31]. On the other hand, some pathogenic bacteria secrete PI metabolising enzymes, like kinases and phosphatases, that they use to subvert PI signalling in the host cells, promoting virulence and survival in the infected cells [9,10]. Although the biological role of the ALPs identified in this study remains unknown, it is interesting to note that many of the sequences belong to pathogenic microorganisms, such as *Listeria*, *Yersinia*, *Klebsiella*, *Clostridium, Leishmania, Candida*, and *Aspergillus*.

Phylogenetic analysis showed a number of clusters, distinct for the bacterial and fungal/protozoan species, and that ALPs are clearly different to classic mammalian lipid 3-ptases, MTMs and PTEN or the PFAs. Additionally, ALPs lack the typical lipid anchoring domains commonly found in mammalian lipid phosphatases.

Biochemical characterisation of selected sequences from the intracellular pathogens *L. monocytogenes *and *L. major*, confirmed our prediction that these are active phosphatases, able to dephosphorylate phosphoinositides. A broader PI activity in ALPs is also in contrast with the more restrictive specificity observed in MTMs/PTEN 3-ptases and the capability to dephosphorylate di- and tri-phosphoinositides found in the SopB/IpgD 5-ptases.

Clear differences were observed in the activity profile between the two proteins in *L. monocytogenes*, Lmo1800 and Lmo1935. While Lmo1935 has preference for peptide substrates, Lmo1800 exhibits a similar activity profile as MptpB, with DSP activity and broad PI specificity. This suggests that the two Lmo proteins could have distinct functional roles and provides an explanation for the presence of the two genes in *Listeria*.

The catalytic function of critical P-loop residues in classic eukaryotic lipid phosphatases has previously been determined. In MTMs, position 6 in the P-loop is occupied by an Asp residue (HCSDGW**D**R), which functions as the general acid in catalysis [32]. Similar findings were reported for MptpB [15]. Here we established the importance of the conserved Asp in the ALP proteins as mutation to Ala results in mutant enzymes with compromised activity, suggesting a similar role of this residue in catalysis as shown for MptpB and MTMs.

In PTEN, the presence of several basic residues (HC**K**AG**K**G**R**) facilitates the binding of highly negatively charged PI substrates and in particular of PI(3,4,5)P3, its preferred substrate [29]. A similar motif, C**K**SX**K**DR, is also found in SopB/IpgD inositol and mammalian 4-ptases that have a preference for poly-phosphate PIs. However, ALPs, contain only two basic residues (Lys and Arg) in the P-loop and they dephosphorylate preferably mono-phosphoralyted PIs with no activity towards PI(3,4,5)P3. Here we have shown that firstly, the conserved Lys in position 5 of the P-loop is important for efficient dephosphorylation of PI(3,5)P2, and probably involved in substrate binding, consistent with the proposed role of the cognate Lys164 in MptpB [15] and Lys128 in PTEN [29]. Secondly, we showed that the number of basic residues is critical to dephosphorylate poly-phosphate PIs. We mutated the residues in position 2 of the P-loop to Lys to mimic the extra basic residue in PTEN and SopB. Mutation of Phe161 in MptpB, Thr190 in Lmo1800 and Thr148 in LM1 to Lys resulted in enhanced activity towards mono- and di-phospho PIs in Lmo1800, and MptpB, and new activity towards PI(3,4,5)P3 for MptpB (Figure 5B). This is an important finding as, historically, the role of P-loop residues has been restricted to enzyme catalysis and catalytic efficiency. In this study we demonstrate that single amino acid substitutions can result in subtle changes in substrate preferences and supports the notion that the active site loop sequence can be used as diagnostic of substrate specificity. New insights regarding the structural constrains that determine substrate binding in this new family of phosphatases should be revealed when the three-dimensional structures are determined.

## Conclusions

Overall, this new family of enzymes shows both distinct sequence features and biochemical characteristics. ALPs have a unique P-loop signature and exhibit broad PI specificity. In addition, they have low sequence homology and different domain organisation to classic lipid phosphatases. The biological role of these proteins remains to be elucidated, but their potential role in PI metabolism suggests interesting links with established mechanisms of pathogenesis in other microorganisms such as *M. tuberculosis, Leishmania*, *Listeria, Salmonella*, and *Shigella *[9,33], and a potential to be exploited as targets in anti-infective treatments given their low homology to any other human phosphatases.

## Methods

### Bioinformatics analyses

Two approaches were taken to identify MptpB related sequences, a) Blast searches [34] of SwissProt and TrEMBL using the full length sequence of MptpB and b) ScanProsite search of SwissProt and TrEMBL http://www.expasy.ch/tools/scanprosite using the signature motif "HCXXGKDR". In the Blast searches the sequences selected had E-values between 10^-11 ^and 10^-131^(> 30% sequence identity). Multiple sequence alignments of MptpB-related phosphatases were performed using ClustalX [35] and manually edited using the programs BioEdit (Hall T: Bioedit http://www.mbio.ncsu.edu/BioEdit/bioedit.html) and Cinema [36]. Phylogenetic trees were calculated using a maximum likelihood program, RAxML [37], with a 100 bootstrap replicates. Trees were produced using the program Treeview [38]. Analysis of the domain architecture was preformed using InterProScan [39] and SMART databases [40].

### Over-expression and purification of recombinant proteins

All the proteins used in the biochemical characterisation were produced in *E.coli *as recombinant proteins as described below:

#### MptpB

The open reading frame of Rv0153c, encoding MptpB, was amplified from *M. tuberculosis *H37Rv DNA and cloned into a pET28a vector (Novagen) to generate an N-terminal His_6_-tagged expression construct. Site directed mutagenesis of the following residues; F161A, F161K, K164A and D165N were carried out using the QuikChange kit (Stratagene). Each construct (WT and mutants) were transformed into *E. coli *BL21 (DE3) and expression was induced using an auto-induction method at 25°C for 16 h [41]. His-tagged MptpB WT and mutants was purified by standard nickel affinity chromatography on a 5 ml HiTrap column (Amersham Bioscience) in binding buffer (50 mM Hepes, 500 mM NaCl, pH 7) and eluted 300 mM imidazole.

#### Lmo1800 and Lmo1935

The open reading frame of *Lmo1800 *and *Lmo1935*, encoding Lmo1800 and Lmo1935 respectively, was amplified from *Listeria monocytogenes *EGD-e DNA and cloned into pGEX-6P-1 (GE Healthcare) to generate an N-terminal glutathione S-transferase (GST) tagged expression construct. Site directed mutagenesis of the following residues; T190K, K193A and D194A (for Lmo1800) and D220A for Lmo1935 were carried out using QuikChange. Each construct (WT and mutants) was transformed into *E. coli *BL21 and expressed using an auto-induction method at 25°C for 16 h [41]. The expressed GST-Lmo1800 and GST-Lmo1935 were purified by glutathione sepharose affinity chromatography. The supernatant from the bacterial lysate was loaded onto a 1 ml GST Trap HP column (GE Healthcare) in binding buffer (50 mM Hepes, 500 mM NaCl, pH 7) and the protein was recovered following on-column cleavage of the GST-tag with PreScission protease (GE Healthcare). For Initial activity assays we used tag-less proteins (Lmo1800 WT and Lmo1935 WT) against phospho-peptides and PIs. Subsequent assays used GST-tag proteins (WT and mutants of Lmo1800 and Lmo1935) following elution with 10 mM glutathione of the glutathione sepharose column. No significant differences in activity were observed for the fusion proteins.

#### LM1

The *Leishmania major *LmjF22.0250 (SP code Q4QBX1) initial pET14 construct was a kind gift from Wesley Van Voorhis, Fred Buckner and Erin Quartley of the Structural Genomics of Pathogenic Protozoa consortium. This construct contained a frame shift, which was subsequently removed by site directed mutagenesis. Site directed mutagenesis of the following residues; T148K, K151A and D152A were carried out using QuikChange. Each construct (WT and mutants) were transformed into *E. coli *BL21 (DE3) and expression was induced using an auto-induction method at 25°C for 16 h [41]. The expressed His_6_-LM1 WT and mutants were purified by nickel affinity chromatography. The supernatant from the bacterial lysate was loaded onto a 1 ml HiTrap column (Amersham Bioscience) in binding buffer (50 mM Hepes, 500 mM NaCl, pH 7) and the protein was eluted with 300 mM imidazole.

#### TbPTP1

*Tb*PTP1 was amplified from *T. brucei *DNA and cloned into pET28a (Novagen) as previously reported [42,15]. Recombinant *Tb*PTP1 was expressed in *E. coli *strain BL21 DE3 Codon+ RIPL, grown in LB broth at 37°C and induced at 30°C with 0.4 mM IPTG. Purification of His-tagged *Tb*PTP1 was performed by nickel affinity chromatography using the same method as for MptpB.

### Phosphatase activity assays

The malachite green assay [43] was used to determine the amount of free phosphate during the dephosphorylation assays with a range of substrates: phospho-Tyr peptides from EGFR (DADEpYLIPQQG) and insulin receptor (TRDIpYETDYYRK), phospho-Ser peptide (RRApSVA), phospho-Thr peptide (KRpTIRR) (Alta Bioscience, University of Birmingham, http://www.altabioscience.bham.ac.uk), pNPP (Sigma), and the PIs diC8-PI(3)P, diC8-PI(3,4)P2, diC8-PI(3,5)P2, diC8-PI(4)P, diC8-PI(4,5)P2, diC8-PI(5)P and diC8-PI(3,4,5)P3 (Echelon Bioscience), phospho-amino acids, adenosine 5' monophosphate, inosine 5'-monophosphophate, phosphorylcholine chloride, phosphorylethanolamine, glycerol 2-phosphate and sodium pyrophosphate (Sigma). Each reaction was prepared in triplicates in a 96-well microplate, containing 50 μl of reaction mix with 5 μg enzyme (MptpB, Lmo1800, Lmo1935, LM1) or 20 μg GST-Lmo enzymes in buffer (50 mM Tris, 50 mM BisTris, 100 mM sodium acetate, pH 6) and 100-125 μM substrate. The reactions were incubated for 15 minutes at 37°C prior to the addition of 50 μl malachite green reagent (Sigma) and further incubated for 10 minutes at room temperature. The absorbance was subsequently read at 620 nm and the mean calculated. Control reactions containing no enzyme were included to measure the background level of phosphate. A phosphate standard curve was produced using known amounts of phosphate (25-3000 pmoles of Sigma phosphate standard solution). Experimental points were interpolated in the standard curve to calculate the amount of phosphate released, which was then used to calculate the specific activity (SA, nmoles min^-1 ^mg^-1^).

## Abbreviations

PI: phosphoinositide; 3-PTASES: inostitol 3-phosphatases; 4-PTASES: inostitol 4-phosphatases; 5-PTASES: inostitol 5-phosphatases; DSP: dual-specificity phosphatase; PTP: protein tyrosine phosphatase; ALP: atypical lipid phosphatase; P-TYR: phosphotyrosine; P-SER: phosphoserine; P-THR: phosphothreonine; PTEN: phosphatase and tensin homologue deleted on chromosome 10; PNPP: *p*-nitrophenol phosphate; EGFR: epidermal growth factor receptor.

## Authors' contributions

NB designed the experiments, produced the data, analysed the results and wrote the manuscript, CS produced reagents and part of the data, HB produced reagents and part of the data, IR supervised part of the work and edited the manuscript, LT designed the experiments, analysed the data and wrote the manuscript. All authors have read and approved the final manuscript.

## Supplementary Material

Additional file 1 **Full multi-sequence alignment of bacterial ALPs**. Alignment of MptpB related sequences identified from a Blast analysis highlights that the sequences are highly divergent except from the phosphate-binding loop (P-loop) in the active site region with an extended signature (C4). There are additional regions of high conservation (labelled C1-C5). Conservation is indicated, "*" 100% conservation, ":" > 75% conservation and "." > 50% conservation. Insertions 1 and 2 are unique to the mycobacteria sequences and labelled in black. Consensus sequences for the conserved regions are indicated on the top row. Alignment prepared using ClustalX [35]. Click here for file

Additional file 2 **List of sequences used in the phylogenetic tree analysis**. Click here for file
