# Supplementary figures and images for: A new family of phosphoinositide phosphatases in microorganisms: identification and biochemical analysis
Source: BMC Genomics. 2010 Aug 2;11:457. doi: 10.1186/1471-2164-11-457 (PMC3091653; doi:10.1186/1471-2164-11-457)

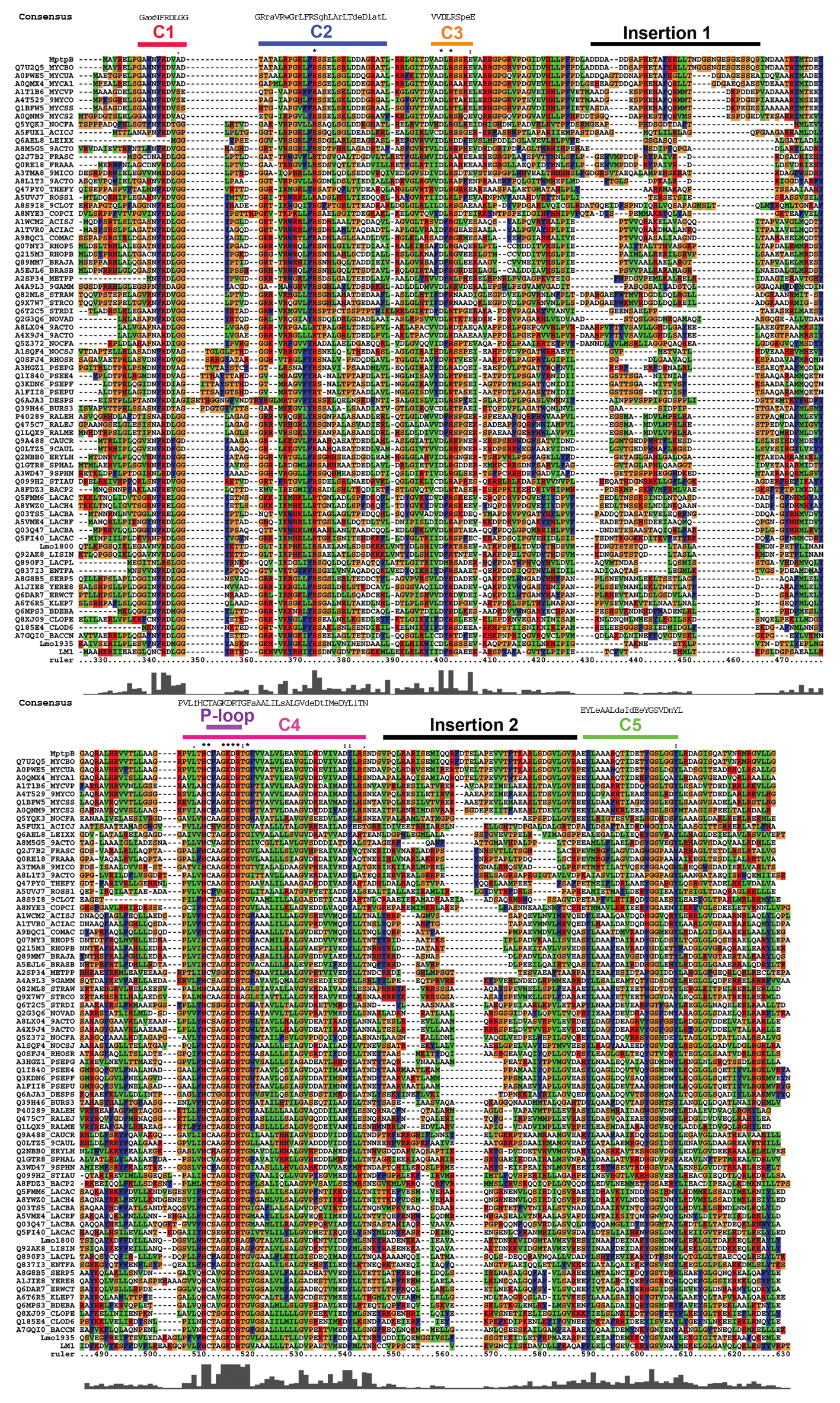

Supplement: Additional file 1 — Full multi-sequence alignment of bacterial ALPs. Alignment of MptpB related sequences identified from a Blast analysis highlights that the sequences are highly divergent except from the phosphate-binding loop (P-loop) in the active site region with an extended signature (C4). There are additional regions of high conservation (labelled C1-C5). Conservation is indicated, "*" 100% conservation, ":" > 75% conservation and "." > 50% conservation. Insertions 1 and 2 are unique to the mycobacteria sequences and labelled in black. Consensus sequences for the conserved regions are indicated on the top row. Alignment prepared using ClustalX [35]. [file 1471-2164-11-457-S1.JPEG]
